# A Common Practice of Widespread Antimicrobial Use in Horse Production Promotes Multi-Drug Resistance

**DOI:** 10.1038/s41598-020-57479-9

**Published:** 2020-01-22

**Authors:** S. Álvarez–Narváez, L. J. Berghaus, E. R. A. Morris, J. M. Willingham-Lane, N. M. Slovis, S. Giguere, N. D. Cohen

**Affiliations:** 1 Department of Large Animal Medicine, College of Veterinary Medicine, University of Georgia, Athens, Ga USA; 2 Department of Large Animal Clinical Sciences, College of Veterinary Medicine & Biomedical Sciences, Texas A&M University, College Station, TX USA; 3 Hagyard Equine Medical Institute, Lexington, KY USA

**Keywords:** Metagenomics, Bacterial genetics, Bacterial infection, Clinical microbiology, Metagenomics

## Abstract

The practice of prophylactic administration of a macrolide antimicrobial with rifampin (MaR) to apparently healthy foals with pulmonary lesions identified by thoracic ultrasonography (i.e., subclinically pneumonic foals) is common in the United States. The practice has been associated epidemiologically with emergence of *R. equi* resistant to MaR. Here, we report direct evidence of multi-drug resistance among foals treated with MaR. *In silico* and *in vitro* analysis of the fecal microbiome and resistome of 38 subclinically pneumonic foals treated with either MaR (n = 19) or gallium maltolate (GaM; n = 19) and 19 untreated controls was performed. Treatment with MaR, but not GaM, significantly decreased fecal microbiota abundance and diversity, and expanded the abundance and diversity of antimicrobial resistance genes in feces. Soil plots experimentally infected with *Rhodococcus equi* (*R. equi*) and treated with MaR selected for MaR-resistant *R. equi*, whereas MaR-susceptible *R. equi* out-competed resistant isolates in GaM-treated or untreated plots. Our results indicate that MaR use promotes multi-drug resistance in *R. equi* and commensals that are shed into their environment where they can persist and potentially infect or colonize horses and other animals.

## Introduction

Despite their beneficial properties for treating infections, injudicious antimicrobial use can promote resistance in bacteria in both human and animal populations^1,2^. The United States leads the list of high-income countries for antibiotic consumption, with ~80% of its annual antimicrobial consumption used for treating disease or promoting growth in animals^3,4^. Furthermore, several studies suggest that the use of antibiotics in animals contributes to the crisis of antibiotic-resistant infections in humans^5–7^.

Although antimicrobial use in food animal production has received considerable attention^8–14^, antimicrobial resistance in equine medicine has received relatively limited attention. Pneumonia caused by *Rhodococcus equi* (*R. equi*) in foals is an important problem for the equine breeding industry worldwide^15–17^. Foals are exposed to *R. equi* from birth^18,19^, and the disease generally progresses insidiously with onset of signs typically between ages 1 and 5 months^19,20^. The combination of a macrolide with rifampin has been the standard of care for foals infected with *R. equi* in North America for over 30 years^21^. Because of its insidious onset, many farms in North America have implemented serial thoracic ultrasonographic screening of foals to identify foals with pneumonia prior to the onset of clinical signs (*i.e*., subclinical pneumonia) and treatment of subclinical pneumonia^21–23^. Evidence exists that most foals with subclinical pneumonia attributed to *R. equi* will not develop clinical signs of pneumonia^21,24,25^. Consequently, thoracic ultrasonographic screening combined with antimicrobial treatment of foals with subclinical pneumonia results in overuse of antimicrobials.

Resistance to MaR in *R. equi* has been increasing in prevalence over recent years in central Kentucky^26,27^. Recently, MaR-resistant *R. equi* (**MRRE**) were identified in environmental samples from 76 of 100 horse-breeding farms in central Kentucky^17^. Resistance to macrolides in *R. equi* has been attributed to a novel transferable *erm* gene (*erm*(46)) that also confers resistance to lincosamides and streptogramins B^28,29^. Observational studies have linked the emergence and environmental burden of MRRE at horse-breeding farms to increased use of MaR (as a consequence of mass antimicrobial preventive treatment of subclinical pneumonia)^17,30^. Evidence is lacking of direct effects of treatment on antimicrobial resistance in individual foals, however.

The emergence of and spread of MRRE calls for alternatives to MaR for treating foals. Many conventional antimicrobials are ineffective or ill-suited for use in foals (*e.g*., vancomycin is effective against MRRE but its use should be reserved for needs of human medicine)^31–35^. Gallium maltolate (**GaM**) is a metal-based compound with *in vitro* and *in vivo* antimicrobial activity against *R. equi*, including MRRE^36–39^. Use of GaM as an alternative to MaR might reduce emergence of MRRE in equine populations and the potential for downstream harm to humans. Recently, we demonstrated that GaM was non-inferior to MaR for treating subclinical pneumonia in foals^32,38^. Although GaM treatment in foals appears to be safe^40^, the impact of GaM on the diversity of the fecal microbiota is unknown.

The purposes of the study were to directly observe and compare the impact of treatment with MaR or GaM in individual foals with subclinical pneumonia – and untreated, age-matched, healthy foals without subclinical pneumonia – on: (i) the diversity of the gastrointestinal tract (GIT) microbiota, (ii) the abundance and diversity of antimicrobial resistance genes (ARGs) of GIT bacteria, (iii) the number of *R. equi* (the pathogen targeted for treatment) and *Enterococccus* spp. (selected as a marker organism because it is a common genus in foal feces) isolates in fecal swabs from foals and antimicrobial susceptibilities of these isolates. Because foals excrete fecal bacteria and unabsorbed antimicrobials and antimicrobial metabolites (via bile) in their feces, an additional objective of this study was to examine how the persistence of and selection for MRRE in the natural environment (soil) was impacted by treatment with MaR and GaM. We provide direct evidence that the common practice^41^ of orally treating foals with subclinical pneumonia with MaR results in dysbiosis, increased the abundance and diversity of resistance genes in their fecal bacteria, and selected for bacteria resistant to MaR and other antimicrobials. Moreover, we demonstrate that exposure to a macrolide promoted the survival of macrolide-resistant *R. equi* in experimentally-infected soil plots. Notably, these changes to fecal bacteria and environmental selection observed for macrolides were not observed for GaM.

## Results

### MaR treatment alters the GIT microbiota and increases the abundance and diversity of genes associated with resistance to macrolides and other antimicrobials

#### Analysis of the GIT microbiota

The diversity of the GIT microbiota among foals treated for subclinical *R. equi* pneumonia with either standard treatment (MaR; n = 19 foals) or GaM (n = 19) were compared to untreated controls (n = 19), before and 14 days after treatment. Fecal samples from these 57 foals collected before and after treatment (114 samples total) were subjected to PCR amplification and subsequent sequencing of the 16S rNA gene V4 region. A total of 11,099,952 reads with an average length of 250 bp were obtained (median, 48,633 reads per sample) totaling approximately 2.75 Gb. The filtering process retained 7,794,828 reads (median, 34,290 reads per sample) with an average length of 253 bp (*N.B*., the length of the 16S V4 region is approximately 250 bp). These reads translated into more than 500,000 amplicon sequence variants (ASVs) per treatment group and time-point (Fig. S1.A) that were ultimately used to measure taxa diversity and abundance. The median number of reads before treatment were 34,097, 33,701, and 34,557 for the control, GaM, and MaR groups, respectively. The median number of reads after treatment were 34,735, 34,700, and 33,781 for the control, GaM, and MaR groups, respectively.

**Figure 1:**
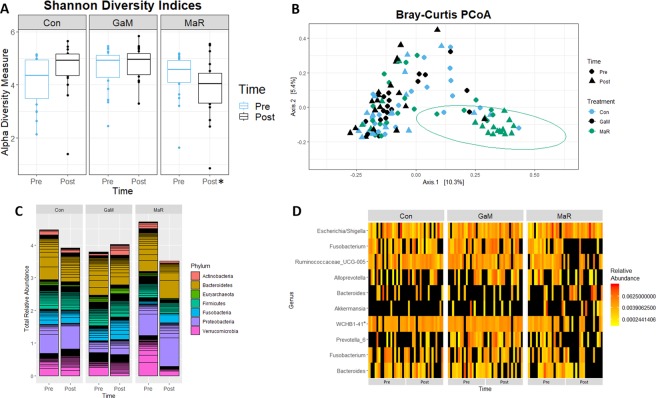
Treatment with macrolides and rifampin (MaR) significantly reduced the abundance and diversity of the gastrointestinal tract (GIT) microbiota. Plots represent data before and after treatment for 3 groups of foals: foals treated with either gallium maltolate (GaM) or MaR and the untreated control group (Con). (**A**) Boxplot of Shannon diversity indices. Asterisk denotes statistical significance (*P* < 0.05). Horizontal lines bisecting boxes represent the median, and the tops and bottoms of the boxes represent the 75^th^ and 25^th^ percentiles, respectively; vertical lines extend to a multiple of 1.5 of the respective interquartile range. Statistical significance was determined using linear mixed-effects modeling with post hoc pair-wise testing using the method of Sidak. (**B**) Bray-Curtis dissimilarity principal coordinate analysis (PCoA) plot. Green circle represents the 95% confidence elipse and corresponds to a significant (*P* < 0.05) clustering of the MaR samples after treatment. Statistical significance was determined by permutation multivariate analysis of variance. (**C**) Stacked bar graph of the total relative abundance of the top 7 phyla represented as a proportion of observed amplicon sequence variants (ASVs) relative to the total number of ASVs. (**D**) Heatmap of the relative abundance of the top 10 ASVs (represented at the genus level) of the GIT microbiota; * WCHBI-41 (4^th^ row from the bottom of the figure) represents the class level; yellow represents lower abundance, red represents higher abundance, and black denotes an absence of abundance.

Before treatment, there were no significant differences in the diversity or abundance of microbes among the 3 groups, but after treatment, the diversity changed and overall microbial abundance decreased for MaR-treated foals (Fig. 1). Values of the Shannon diversity index decreased significantly (*P* < 0.05) among MaR-treated foals, but did not decrease for GaM-treated or control foals (Fig. 1A). Similar results were seen for the observed ASV measure of richness of α diversity (Fig. S1.B). Results of principal coordinates analysis (PCoA) of Bray-Curtis dissimilarity indices indicated a significant (*P* < 0.05) clustering of the post-treatment samples from the MaR-treated foals (Fig. 1B).

The 20 most-represented taxa (Fig. S2) were evaluated for treatment-driven changes in the GIT microbiota diversity. Bacterial communities in all samples were comprised primarily of ASVs belonging to 7 phyla (Fig. 1C). Independent of treatment and time-point, Bacteroidetes was the predominant phylum followed by Proteobacteria, Verrucomicrobia. Firmicutes and Fusobacteria. Actinobacteria and Euryarchaeota were the least represented phyla (Fig. 1C). The diversity of phyla changed and the relative bacterial abundance (*i.e*., the proportion of the observed ASVs to the total number of ASVs) was lower after treatment with MaR, relative either to MaR foals before treatment or to foals in the other groups before or after treatment (Fig. 1D). All phyla except Proteobacteria were altered among the MaR-treated foals.

#### ARGs analysis

The GIT resistome was studied to determine if MaR or GaM treatment expanded the abundance or diversity of antimicrobioal resistance ARGs. Sequencing generated a total of 32,936,952 reads (median, 254,247 reads per sample), of which 446,500 were associated with antibiotic resistance genes. Two samples were excluded from the analysis: 1 pre-treatment sample from a MaR group foal for which bacterial DNA concentration was insufficient for sequencing, and 1 post-treatment sample from a foal in the MaR group that yielded only 33,090 reads (8-fold below the average) with no hits for ARGs above the 80% similarity threshold.

**Figure 2:**
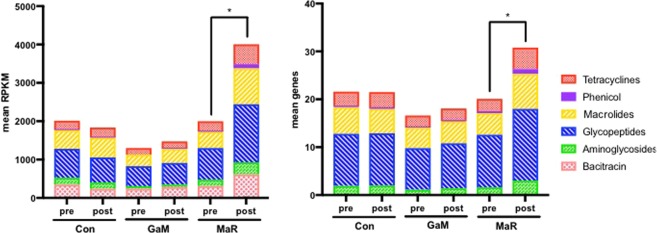
Treatment with MaR resulted in a significant (*P* < 0.05) increase in the abundance (represented as reads per kilobase of transcript per million mapped reads [RPKM]) and diversity of genes encoding resistance to 6 classes of antimicrobials. Plots represent data before and after treatment for 3 groups of foals: gallium maltolate (GaM), macrolide and rifampin (MaR), and the untreated control group (Con). Asterisk denotes statistical significance determined using linear mixed-effects modeling with post hoc testing of pair-wise differences using the method of Sidak^83^ (*P* < 0.05). See Fig. S4 for more details regarding the statistical analysis.

The ARG abundance expressed in RPKMs (reads per kilobase of transcript, per million mapped reads) and ARG diversity expressed as the number of ARGs identified were compared before and after the treatment period. MaR treatment significantly (*P* < 0.05) increased RPKM counts after 14 days of treatment, whereas no change was observed after treatment for either the GaM or control foals (Fig. S3). No significant changes were observed in total ARG diversity among any of the treatment groups or time-points (Fig. S3). However, the feces of MaR-treated foals had a significant (*P* < 0.05) increase in RPKM counts and diversity of ARGs encoding resistance to aminoglycosides, glycopeptides, macrolides, phenicols, and tetracycline (Figs. 2, S4). A significant increase of RPKM counts that was not associated with an increase in diversity of ARGs for bacitracin resistance also was observed after MaR treatment (Figs. 2, S4). Treatment with GaM did not alter the total RPKM counts or diversity of ARGs (Figs. 2, S4). We observed for the MaR-treated foals a significant (*P* < 0.05) increase in RPKM counts and diversity of ARGs to: aminoglycosides (inactivation enzymes, O-phosphotransferase, and O-nucleotidyltransferase); bacitracin (ABC transporters); glycopeptides (Van-type regulator proteins), macrolides (inactivation enzymes); phenicol (acetyltransferases), and tetracycline (ribosomal protection proteins) (Fig. S5). Similarly, a significant (*P* < 0.05) increase of RPKM counts, but *not* diversity of ARGs was observed in MaR-treated foals for: glycopeptides (Van-resistance proteins and Van-accesory proteins); and macrolides (macrolide efflux-pumps and ribosomal protection methyltransferases) (Fig. S5).

### MaR treatment increases resistance to macrolides and other antimicrobials in fecal bacteria relative to controls

**Figure 3:**
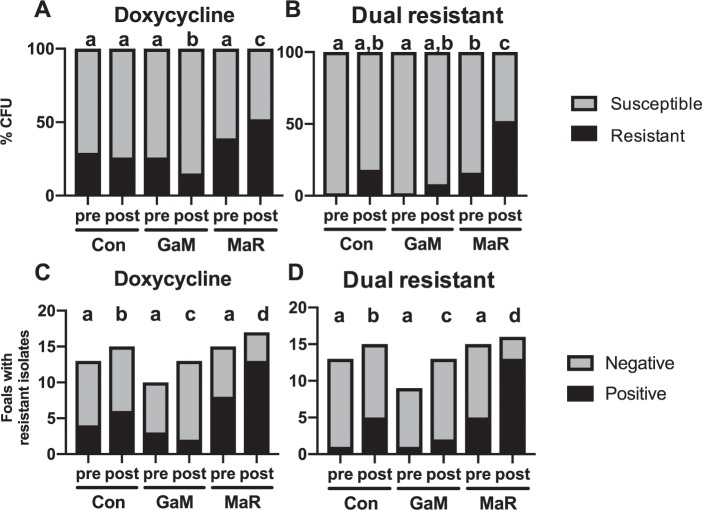
Treatment with macrolides and rifampin (MaR) increased the proportion of fecal *Enterococcus* spp. isolates resistant to doxycycline or to both doxycycline and erythromycin and the number of foals shedding these isolates in feces. Bar charts represent the percentage of enterococcal isolates either resistant (black) or susceptible (grey) to: (**A**) doxycycline, (**B**) doxycycline and erythromycin (dual resistant). Bar charts representing the number of foals shedding enterococcal isolates resistant to either (**C**) doxycycline or (**D**) to both doxycycline and macrolides (dual-resistant). Data were analyzed using linear mixed-effects modeling with post hoc pair-wise testing using the method of Sidak^83^; bars with different letters differ significantly (*P* < 0.05).

**Figure 4:**
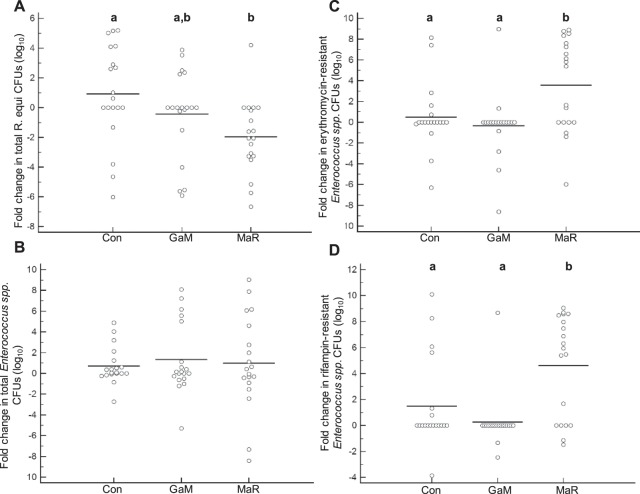
Treatment with macrolides and rifampin (MaR) significantly decreased fecal shedding of *R. equi* in affected foals but promoted a significant increase in the number of macrolide-resistant and rifampin-resistant *Enterococcus* spp. Dot plots present bacterial fold-changes by treatment group for: (**A**) total colonies of *R. equi*, (**B**) total colonies of *Enterococcus* spp., (**C**) colonies of erythromycin-resistant *Enterococcus* spp., and (**D**) colonies of rifampin-resistant *Enterococcus* spp. Con = untreated control group; GaM = gallium maltolate. Horizontal lines represent sample means. Groups with different letters differ significantly (*P* < 0.05) as determined using linear mixed-effects modeling with post hoc pair-wise comparisons made using the method of Sidak^83^; no significant differences were detected in data represented in panel B.

The impact of treatment on fecal bacterial populations and selection for resistant bacteria was further examined by assessing antimicrobial resistance phenotype among fecal isolates of *R. equi* (the pathogen targeted for treatment) and *Enterococccus* spp. (selected as a marker organism because it is a common genus in foal feces) that were collected before and after treatment (Fig. 3). Treatment with MaR significantly (*P* < 0.05) decreased the number of *R. equi* isolates obtained from fecal swabs compared to untreated control foals (Fig. 4A), whereas GaM did not. During the course of the study, only 1 macrolide-resistant and 2 rifampin-resistant isolates of *R. equi* were isolated from a fecal swab of a single foal post treatment with MaR, precluding meaningful comparison of treatments on frequency of resistance to these antimicrobials. Treatment with MaR did not reduce shedding of *Enterococcus* spp. (Fig. 4B). Furthermore, there was a significant (*P* < 0.05) increase in macrolide-resistant (Fig. 4C) and rifampin-resistant (Fig. 4D) *Enterococcus* spp. after 2 weeks of treatment with MaR.

Our *in silico* results indicated that MaR treatment decreased the number and diversity of bacteria colonizing the foal’s GIT while selecting for microbes that were intrinsically resistant or that acquired resistance to *several* classes of antibiotics. To substantiate these *in silico* findings, we performed antimicrobial phenotypic profiling of 1,140 *Enterococcus* spp. fecal colonies isolated from foals to the selected antimicrobials identified in our resistome analysis. We evaluated the effects of treatment on resistance of *Enterococcus* spp. using either individual *Enterococcus* spp. isolates or foals as the unit of analysis. Using *Enterococcus* spp. as the unit of analysis, resistance to macrolides, rifampin, or doxycycline did not differ significantly before treatment, but after treatment resistance to these antimicrobials rose slightly but significantly (*P* < 0.05) in the MaR-treated foals (Figs. 3; S7). In contrast, the proportion of isolates resistant to these 3 antimicrobials decreased significantly for the GaM group (Figs. 3; S7). The proportion of dual-resistant isolates significantly increased over time, and there was a significantly higher proportion of dual-resistant *Enterococcus* spp. in the MaR-treated group compared to the other groups both before and after treatment (Fig. 3B). To corroborate the bacterial culture and susceptibility results (phenotypic data) with our previous *in silico* resistome analysis (genotypic data), we examined treatment-driven changes in *Enterococcus* spp. RPKM counts and diversity of ARGs. Only 11 enterococcal genes in the resistome database were associated with MLS resistance (3 genes were identified in *Enterococcus faecalis* and 8 in *Enterococcus faecium*), and 1 gene with tetracycline resistance (tetU, *Enterococcus faecium*). Rifampin resistance is generally caused by a punctual mutation in the *RpoB* gene and therefore it was not represented in our resistome database and not possible for us to examine. We found a significant (*P* < 0.05) increase in both RPKMs and diversity of ARGs for macrolide and tetracycline resistance but not for the other groups (Fig. S8).

### Soil treated with MaR selects for MaR resistance over time

**Figure 5:**
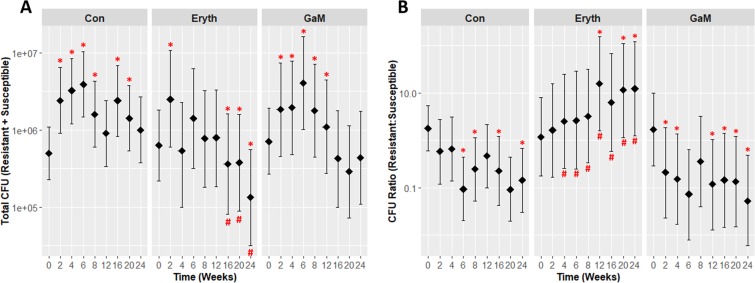
Treatment of soil with a macrolide (erythromycin) significantly decreased the total concentration of *R. equi* but significanlty selected for macrolide-resistant *R. equi*. Means (black diamonds) and 95% confidence intervals (vertical bars) for (**A**) total *R. equi* (colony forming units [CFU] represented on a log_10_ scale) and (**B**) ratios of CFUs of macrolide-resistant to macrolide-susceptible isolatesof *R. equi*. Soil samples were serially cultured quantitatively during a period of 24 weeks. Red pound symbols indicate significant (*P* < 0.05) differences among treatments within time, and red asterisks indicate significant (*P* < 0.05) differences among times relative to week 0 (before treatment) within treatment. Data were analyzed using linear mixed-effects modeling with post hoc pair-wise testing using the method of Sidak^83,84^.

Foals excrete fecal bacteria into their environment and a variable fraction of any oral antimicrobials administered that are unabsorbed or circulated entero-hepatically. In foals, macrolides commonly used for treating *R. equi* pneumonia have bioavailabilities of ≤50%, and these drugs and their metabolites can be excreted in feces^42,43^. Thus, we postulated that macrolide-treated foals excrete into their environment both macrolide-resistant bacteria and compounds (unabsorbed macrolides and their metabolites) that favor survival of these resistant organisms. To test this hypothesis, we infected soil plots with 2 isogenic strains of *R. equi* that differed only in the presence of the macrolide resistance element *erm*(46) at a ratio of 1:1, then treated plots with MaR, GaM, or nothing (controls), and monitored growth of the isogenic strains over a 24-week period. Significant (*P* < 0.05) effects of treatment, time, and their interaction on the total CFU of *R. equi* in soil plots were noted (Fig. 5). In the untreated plots, the total *R. equi* concentration increased significantly (*P* < 0.05) by week 2 and remained significantly (*P* < 0.05) higher than week 0 at all time-points except weeks 12 and 24 (Fig. 5A). The proportion of macrolide-resistant isolates in the control plots, however, decreased continuously over time, becoming significantly (*P* < 0.05) lower than baseline from weeks 6 through 24(Fig. 5B). In the MaR-treated plots, the total concentration of *R. equi* initially increased significantly (*P* < 0.05) at week 2, and then began to decrease, becoming significantly lower (*P* < 0.05) than the concentration at week 0 from weeks 16 through 24 (Fig. 5A). The proportion of macrolide-resistant isolates in the MaR-treated plots, however, increased over time. By week 16, MaR-resistant *R. equi* had out-competed the susceptible isogenic strain: there was an approximately 10-fold increase in concentration of resistant isolates relative to susceptible *R. equi*. Moreover, from weeks 4 to 24, the proportion of MaR-resistant isolates found in MaR-treated soil was significantly (*P* < 0.05) higher than either control or GaM-treated plots at each corresponding time-point (Fig. 5B). In the GaM-treated plots, concentrations of total *R. equi* significantly (*P* < 0.05) increased after 2 weeks, and peaked at week 6 before beginning to decrease (Fig. 5A). By week 16, total *R. equi* in GaM-treated soil had decreased to concentrations comparable to those observed at week 0. The proportion of macrolide -resistant isolates in GaM-treated soil had significantly decreased relative to baseline (week 0) by week 4 and remained significantly (*P* < 0.05) lower than baseline through week 24 (Fig. 5B).

## Discussion

Pneumonia caused by *R. equi* is a major health problem for equine industries on a global basis^21^. In the absence of highly effective preventatives, the combination of a macrolide and rifampin (MaR) has been routinely used at many horse breeding farms to treat foals with *R. equi* subclinical pneumonia^41^. Evidence exists, however, that most foals with subclinical lesions are likely to resolve without progressing to clinical disease^44^. Thus, the practice of treating subclinically infected foals leads to over-use of MaR. Importantly, this practice has been linked to the development and spread of macrolide- and rifampin-resistant isolates of *R. equi*^27,30^. Here, we show that this practice results in alteration of the fecal microbiota diversity including selection for microbes with genes encoding resistance to not only macrolides but also to multiple other classes of antimicrobials.

As previously reported^45–48^, Bacteroidetes, Firmicutes, Proteobacetria, and Verrucomicrobia were the predominant phyla in foal feces, irrespective of treatment group or sample time-point. Subclinical pneumonia did not appear to impact the GIT microbiota because there were no significant differences before antibiotic treatment between the healthy (control) foals and the foals with subclinical pneumonia. Treatment with MaR decreased the taxa abundance and diversity of the most common bacterial phyla, except for Proteobacteria which increased after MaR treatment. Proteobacteria of human neonates treated with macrolides have yielded conflicting results, suggesting that effects on the human GIT microbiota may be specific to the age and geographic setting of its recipients^49,50^. To our knowledge, the only published report of the effects of rifampin on the microbiota was conducted using cockroaches and showed that rifampin produced a substantial shift of the microbiota diversity after 10 days of continuous administration. Interestingly, the phylum Proteobacteria was not affected by treatment^51^. Macrolides are generally used to treat respiratory infections caused by gram-positive bacteria and have limited efficacy against gram-negative bacteria^52^. Rifampin is primarily used to treat mycobacterial infections such as tuberculosis in humans or *R. equi* infection in horses^41,53^. Thus, it is not surprising to see that MaR treatment did not have an impact on Proteobacteria, a phylum that is comprised of a wide variety of gram-negative enteric bacteria such as *Escherichia* (commonly found in horses) that are naturally resistant to macrolides and rifampin^54^. We observed that MaR treatment especially reduced the phylum Verrucomicrobia, consistent with reports of a decrease in the taxa abundance and diversity of this phylum in the GIT microbiota of humans treated with antibiotics^49,55^. Furthermore, oral administration of trimethoprim sulfadiazine to horses for 5 days significantly reduced the relative abundance of Verrucomicrobia in the gut of adult mares^56^, suggesting that this phylum (mainly composed by soil and fresh and marine water bacteria) is highly susceptible to antibiotics. Overall, the *in silico* characterization of the foals’ GIT microbiome suggested that MaR would significantly reduce susceptible species (*i.e., R. equi* or phylum Verrucomicrobia) while promoting the growth of naturally resistant commensal bacteria (*i.e*., Proteobacteria). These results were corroborated by our *in vitro* analysis showing that MaR treatment significantly decreased the number of susceptible *R. equi* isolates (as previously reported^57^), but did not reduce the number of *Enterococcus* spp. isolates. Furthermore, we observed that even the genus *Enterococcus* spp. suffered a MaR-driven shift in which MaR-susceptible members were replaced with resistant isolates. These findings also match with previous reports in other animal models. Specifically, tylosin phosphate (a macrolide commonly administered for the control of liver abscesses in cattle) was seen to significantly increase the proportion of macrolide-resistant enterococci by selecting for resistant strains already present within the GIT of cattle^13^.

**Figure 6:**
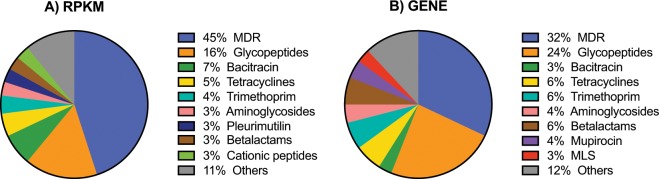
The GIT resistome of healthy foals lacking evidence of subclinical pneumonia. The data presented were obtained from the initial (“pre-treatment”) fecal samples from untreated control foals that lacked evidence of subclinical pneumonia. Pie charts represent the percentage in fecal samples of (**A**) abundance of antimicrobial resistance genes represented as the reads per kilobase of transcript per million reads (RPKM) by class of antimicrobial, or (**B**) diversity of antimicrobial resistance genes by class of antimicrobial.

Results from our untreated control foals represent the first characterization of the fecal resistome in healthy foals (summarized in Fig. 6). The presence of multidrug resistance (MDR) genes in the fecal microbiota of foals likely reflects active contact and ingestion of soil microorganisms and, indirectly, coprophagia of their dams’ feces. A previous analysis of the soil resistome indicated that soil bacteria are rich in multidrug efflux pumps as they are a crucial tool for bacterial survival in the soil ecosystem^58^. Similarly, the mechanisms of resistance to glycopeptides found in our study were mainly *van*-type resistance proteins and regulators previously found in *Streptococcus* spp. and *Enterococcus* spp. These 2 genera are part of the phyllum Firmicutes, one of the major phyla of the equine intestinal microbiota^45–48^. Thus, the observed higher abundance and diversity of glycopeptide resistance genes likely reflects the normal equine fecal microbiota which has a large proportion of Firmicutes.

Our results in foals are similar to those previously observed in humans and other animal species indicating that antimicrobial treatment increases the number of resistant bacteria and the number and diversity of ARGs in fecal bacteria^13,59,60^. Oral treatment with MaR for 14 days significantly increased the overall number of copies of resistance genes but not the diversity of genes related to antimicrobial resistance, possibly suggesting that MaR treatment enriched the intrinsic GIT resistome rather than promoted the acquisition of new ARGs. Although not significant, however, the total number of resistance genes increased in the MaR-treated foals (Fig. S3), suggesting the possibility that variation in the data and our sample size limited to identify this increase as significant. Macrolides are used regularly in food-producing animals to control common diseases^61^ and they have been recently reported to increase ARGs in fecal *Enterococci*^13^. Notably, we observed that MaR treatment significantly increased abundance and diversity of ARGs to multiple antimicrobials (macrolides, aminoglycosides, glycopeptides, phenicols, tetracycline, and bacitracin [abundance only]). The likely explanation for why we saw significantly increased diversity in these classes of antimicrobials but not overall (*i.e*., across all resistance genes) is that resistance genes for these specific antimicrobials represented only a small portion of the total resistance genes (*i.e*., < 6% for each antimicrobial except the glycopeptides). Although we conclude that our results favor enrichment of intrinsic ARGs, further investigation of this subject is warranted.

We documented that our *in silico* data were reflected by phenotypic results. We observed that MaR treatment significantly increased the number of fecal enterococcal organisms resistant to a macrolide (erythromycin) and a tetracycline (doxycycline), and a significantly higher abundance and diversity of enterococcal ARGss for these 2 classes of antimicrobials were found in the feces of the tested foals. We identified 11 enterococcal genes that would confer resistance to macrolides and 1 gene that protects against tetracyclines. The database indicated that these genes belonged to the species *E. feaecalis* and *E. faecium*. Hence, we could hypothesize that these 2 species were the most probable carriers of the ARGs in the fecal samples of our study. Nonetheless, *Enterococcus* spp. is a model genus for studying bacterial horizontal gene transfer because of the facility with which enterococci exchange DNA via conjugation both within the genus and to other genera^62^. Thus, it would not be surprising to find that these particular genes spread to other bacterial genera.

These findings are consistent with previous reports describing development of multi-drug resistance developing in livestock treated with antimicrobials^10,12,14^. A number of mechanistic explanations exist for acquisition of multi-drug resistance, including conjugal transfer of multiple genes encoding resistance to different antimicrobials and multidrug efflux pumps^63^. These findings are of great importance as a cautionary example of the potential consequences of injudicious use of antimicrobials in domestic animals. In considering the results of dual-resistance to macrolides and doxycycline, dual resistance was identified more often in *Enterococcus* spp. isolates from the MaR-treated foals both before and after treatment. The reason for this difference before treatment is unknown, but could have resulted from chance alone or could indicate that the genetic elements encoding resistance to these 2 isolates were linked. Irrespective of this difference at baseline, dual resistance increased only in the MaR treated group. This finding suggests that MaR treatment selectively increased the dual resistant isolates among foals in this group.

We investigated the impact of a macrolide (erythromycin) and GaM in *R. equi* in soil. The rationale for these experiments was that orally administered macrolides are not completely absorbed from the gastrointestinal tract of foals and are thus excreted in feces to their environment^43^. We found that treatment with macrolides induce a selective pressure that selected for macrolide-resistant *R. equi* in the gut and in the environment, and that removing this pressure reduces the proportion of resistant isolates over time. This is likely the result of the resistance genes having a fitness burden for *R. equi*, consistent with other *in vitro* findings from our laboratory^64^. This suggests that removing the selective pressure induced by antibiotics could lead to resistant isolates being out-competed by susceptible isolates. Irrespective of the mechanism of the selection, it is important to note that injudicious use of antimicrobials in domestic animals such as horses has the potential to contaminate environments shared by people and other domestic animals and wildlife with multi-drug resistant pathogens and opportunists (Fig. 4). This is especially important because *R. equi* is a soil saprophyte, and the impact of selecting for macrolide-resistant *R. equi* in the environment on both horizontal gene transfer of these resistance genes and on the health of animals exposed to these resistant organisms remains to be determined.

The problem of resistance creates a great need for alternatives to standard antimicrobials for treating infectious diseases. We have previously demonstrated that GaM is non-inferior to macrolides for treating subclinical pneumonia in foals^38^. Moreover, we have shown that the MIC_90_ of GaM is similar for strains of *R. equi* that are resistant to macrolides and those that are susceptible^36^. Thus, our findings that GaM neither altered the diversity of the fecal microbiota or the fecal resistome, nor favored the persistence of macrolide-resistant *R. equi* in soil is of great clinical relevance. Furthermore, 14 days of treatment with GaM seemed to significantly decrease the number of *Enterococcus* spp. resistant isolates. GaM disrupts bacterial iron metabolism, and interferes with their DNA and protein synthesis^65^. It is conceivable that GaM selectively reduces resistant bacterial populations because they are less fit than susceptible bacteria. Nevertheless, further evaluation of the efficacy and impact on GaM on the resistome of foals is warranted.

This study had a number of limitations. Our sample size was relatively small and was limited to a single geographical region. In addition, we were not able to directly corroborate our *in silico* gene findings with the phenotype of fecal bacteria beyond the *Enterococcus* spp. isolates we examined. We did not evaluate the specific mechanism(s) of multi-drug resistance found in our *Enterococcus* spp. samples, and this would be important to examine in future studies. Last, potential point mutations caused by rifampin were not identified and included in our analysis.

Our findings are important because they demonstrate empirically that widespread use of macrolides and rifampin at horse farms results in propagation of resistance to macrolides in *R. equi* but also resistance in other fecal bacteria to many other antimicrobials. Thus, the widespread use of antimicrobials for treating foals with subclinical pneumonia should be reconsidered because of the potential for increasing and disseminating resistance to multiple antimicrobials. Encouragingly, our soil plot studies indicate that macrolide resistance can be reversed by eliminating selective pressure from administering macrolides. Unfortunately, there are few safe and effective alternatives to macrolides for treating *R. equi*. Further clinical evaluation of GaM is warranted, particularly to determine whether GaM administration can stem the tide of emerging macrolide- and rifampin-resistant *R. equi* resulting from overuse of macrolides in equine practice.

## Materials and Methods

### Experimental design and sample collection

This study was approved by the University of Georgia Institutional Animal Care and Use Committee and the Hagyard Equine Medical Institute. All work was conducted in accordance with relevant guidelines and regulations and without adverse events in any participating foals. Foals from 4 breeding farms in Kentucky that routinely use ultrasonographic screening to identify and treat foals with lungs lesions (*i.e*., areas of lung consolidation or abscess formation detected sonographically) as part of their program to control *R. equi* pneumonia were recruited for this study. Owners or their authorized agents provided informed consent for participation. Thirty-eight foals with findings of subclinical pneumonia (lung lesions identified sonographically as ≥2 cm but <6 cm in maximal diameter in foals without clinical signs of disease) were randomly assigned to 2 treatment groups: 1) a macrolide and rifampin at recommended dosages (n = 19); or, 2) GaM administered at a dose of 30 mg/kg by mouth every 24 h (n = 19) for 14 days. In addition, age-matched healthy foals from the same farms were included as a control group. A sample size of 20 foals per group was selected on the basis of providing >80% power at a significance level of 5% to detect a difference in the proportion of foals having a post-treatment reduction of diversity index in their feces of 50% in the GaM-treated group compared with 95% among the macrolide-treated group. On the basis of our inclusion criteria, we were only able to include 57 foals (19 per group). None of the foals included in this study were previously administered antimicrobial therapy. Sterile cotton swabs pre-moistened with sterile saline were gently inserted approximately 5 to 8 cm into the rectum. Four rectal swabs per foal were collected at day 0 (pre-treatment) and at day 14 (post-treatment): 2 swabs were used for DNA extraction and subsequent microbiota and resistome analysis, and the other 2 swabs were used for bacterial culture.

### DNA extraction

Fecal DNA was extracted with a kit (QIAamp Fast DNA Stool Kit, Qiagen) following the manufacturer’s conditions with the following modifications. First, the cotton portion of the swab was homogenized prior to standard DNA extraction. Second, we used the following conditions: 100 mg of 0.1-/0.5- mm silica beads and 1 ml of the inhibitEX buffer in a bead-beater for 3 cycles of 45 seconds.

### Microbiome analysis

#### Amplification and sequencing of 16S ribosomal RNA

Microbiota diversity was determined by PCR-amplification of the hypervariable V4 region of the 16S rRNA gene using primers 515 F and 806 R^66^. Libraries were generated using a method in which the sequencing adapters and index sequences are incorporated into the PCR primers, and subsequently sequenced on an Illumina MiSeq paired-end platform at Alkek Center for Metagenomics and Microbiome Research at Baylor College of Medicine (US). *Data analysis:* A custom Perl script (Fig. S6) was used to demultiplex samples without quality filtering. Raw paired-end sequences were sorted in R (version 3.5.1; Fig. S6) by sample name to ensure sample matching. The R package DADA2^67^ was used to group forward and reverse sequences separately (based on file identification), and to subsequently filter and trim the reads based on sequence quality scores. This way, forward and reverse reads were truncated at 240 bp and 180 bp, respectively, and all reads with >2 expected errors were removed. A dereplication step was performed to combine all identical sequence reads into a group with their corresponding abundances. DADA2 was used to account for any PCR amplification or sequencing errors. The errors generated were used to infer the ASVs for this dataset. Sequences were then merged using DADA2 and removal of chimeras was performed. Taxonomic assignment was made to the ASVs based on SILVA rRNA database (version 128). Measures of α diversity, β diversity, and phyla abundance metrics were produced using THE Bioconductor package *phyloseq* (version 1.24.2^68^) and visualized with *ggplot2* R package^69^. Statistical analyses were performed using linear mixed-effects for α diversity metrics (Shannon diversity measure) in R package *nlme* (version 3.1.137^70^) and permutational multivariate of analysis of variance using R package *vegan*7 (version 2.5-3^71^) for the β diversity metric (Bray-Curtis dissimilarity). Significance was set at *P* < 0.05.

### ARG profiling

#### DNA sequencing

DNA library preparation (Nextera DNA Library Preparation Kit) and Illumina MiSeq and NextSeq whole genome shotgun sequencing were performed by the University of Georgia’s Georgia Genomics Facility (US). NextSeq sequencing was added to that from MiSeq to provide adequate depth of coverage and quality of reads. *Resistome data analysis:* Paired-end reads were trimmed using TRIMMOMATIC^72^. Forward and reverse trimmed reads were then sorted by name, using an in-house script (Fig. S6) and paired using software PEAR^73^. Fastq files containing trimmed paired-end reads were transformed to fasta format using an in-house script (Fig. S6). MiSeq and NextSeq were combined with an in-house script as described below. Combined paired-end reads were aligned against MEGARes^74^ and CARD^75^ antibiotic databases using DIAMOND^76^: MEGARes and CARD were merged in a unique database and gene duplications were removed with CD-HIT^77^. ResistomeAnalyzer (https://github.com/cdeanj/resistomeanalyzer) was used to obtain the total count of reads that aligned with >80% similarity (nucleotide level) to each of the ARGs in the database. ResistomeAnalyzer also provided an annotation regarding the resistance mechanism and the antibiotic group affected. Reads were normalized per kilobase of gene per million mapped reads (RPKM). The effects of treatment group, sampling time (baseline versus 14 days), and the interaction of treatment group and sampling time on either the number or proportion of resistance genes (macrolide-specific and overall) were assessed using mixed-effects modeling with a significance level of *P* < 0.05.

### Isolation of *enterococcus* spp. and *R. equi* from fecal samples

Two fecal swabs were used for isolation of both susceptible and resistant *Enterococcus* spp. and *R. equi*. One fecal swab was cultured overnight in NANAT *R. equi*-selective broth^78,79^ and one in brain heart infusion (BHI) broth for enrichment. Quantitative bacteriologic culture of the fecal suspensions was performed by plating serial dilutions on modified *Enterococcus*-selective agar (MESA) and modified NANAT *R. equi*-selective agar medium with and without the presence of antibiotics at the following concentrations when required: GaM, 32 µg/mL; erythromycin, 8 µg/mL; or, rifampin, 25 µg/mL. Data were analyzed using mixed-effects linear modeling, with individual foal nested within farm modeled as hierarchical random effects to account for repeated observations and clustering of observations by foal and farm. A significance level of *P* < 0.05 was used.

### Polymerase chain reaction (PCR)

When quantities were sufficient, the identity of 10 randomly selected isolates of *R. equi* and 10 *Enterococcus* spp. isolates were confirmed by PCR as previously described (refs. ^80^ and^81^, respectively). In addition, resistant *R. equi* isolates were screened for the presence of *erm*(46), the gene which confers resistance to macrolides *in R. equi*^28^.

### Resistance profile screening

Ten randomly selected *Enterococcus* spp. colonies isolated from fecal swabs collected from each foal before or after treatment (n = 57 foals x 10 colonies x 2 time-points = 1,140 isolates) were evaluated for growth on plates containing antimicrobials representative of those identified in the resistome analysis. Each *Enterococcus* spp. colony was sub-cultured on BHI plates containing antibiotics at the following concentrations: erythromycin = 8 µg/ml; amikacin = 64 µg/ml; rifampin = 4 µg/ml; vancomycin = 32 µg/ml; doxycycline = 16 µg/ml; florphenicol = 16 µg/ml; and, clindamycin = 4 µg/ml. The concentrations were based on Clinical Laboratory Standard Institute (CLSI)-established resistance breakpoints for *Enterococcus* spp. Isolates that grew on antibiotic-containing plates were considered resistant. To confirm the validity of the screening results, for each drug, antimicrobial susceptibility testing of 20 randomly selected isolates of *Enterococcus* spp. were evaluated as described below using ETEST® strips (bioMerieux, Druham, NC; hereafter referred to as E-test). All *Enterococcus* spp. isolates tested were resistant to amikacin and clindamycin and susceptible to vancomycin. Consequently, these drugs were not considered in statistical analysis. Discordance of results for the screening method and the E-test results were found for florfenicol, so this drug was not evaluated further. Agreement between the screening and the E-test results was 100% for all resistant isolates, and 100%, 85%, and 70%, for erythromycin-, doxycycline-, and rifampin-susceptible isolates, respectively. Rifampin was included in the analysis because this antimicrobial was a component of the MaR treatment.

### Antimicrobial susceptibility testing

*R. equi* and *Enterococcus* spp. inocula were prepared from overnight cultures in tryptic soy agar (TSA) by the direct colony suspension method according to the guidelines established by the CLSI, resulting in the recommended inoculum of ~1 to 5 × 10^5^ CFU as verified by colony counting. The susceptibility profiles of amikacin (aminoglycoside), clarithromycin (macrolide), erythromycin (macrolide), florfenicol (phenicol), clindamycin (lincosamide), doxycycline (tetracycline), rifampin (rifampin), and vancomycin (glycopeptide) were determined by use of E-tests.

### Inoculation of *R. equi* in soil

Five kg of soil were collected from pastures that normally house horses, mixed homogenously, and then placed into 12, 1000-ml sterilized Nalgene beakers (soil plots). Each soil plot held approximately 500 grams (g) of soil. The soil plots were housed in a BSL2 laboratory, where the temperature was held at approximately 22 °C for the duration of 24 weeks. Macrolide-susceptible *R. equi* strain 103, with a zeocin selection cassette and its isogenic derivative, macrolide-resistant *R. equi* 103 with the *erm*(46) plasmid and a zeocin selection cassette were utilized^28^. The 2 strains were both grown in an incubator at 37 °C overnight in BHI broth supplemented with zeocin and/or erythromycin rotating at 175 rpm. The bacterial suspensions were washed with filter sterilized MilliQ H20, and adjust to a final concentration of 3 × 10^7^ CFU/ml. A total volume of 5 ml of that suspension was added to all 12 soil plots. Quantitative culture was performed biweekly for 8 weeks, and then monthly until 24 weeks onto NANAT *R. equi* selective media plates supplemented with zeocin alone (25 µg/ml) or together with erythromycin (4 µg/ml). At each time point of quantitative culture, 1 g of soil was collected from each soil plot and resuspended in 5 ml of filtered-sterilized MilliQ H2O. Then, 10-fold serial dilutions were performed and plated onto the 2 types of NANAT *R. equi* selective media plates. To maintain nutrients in the soil and mimic the external environment, all soil plots received 5 g of feces weekly, as well as, treatment with 1 g of erythromycin, 1 g of gallium maltolate, or no antimicrobial (control plots). We infected soil samples with 10^6^ CFU/cm^2^ of *R. equi* because this is similar to the concentration of bacteria found in the soil of endemic farms^82^. The rationale for including 1 g of erythromycin was that foals were found to excrete up to approximately 1.5 mg/g of erythromycin in feces, such that 1 g is equal to approximately 1.5 pounds (681 g) of foal feces^42^. Because fecal concentrations of GaM in treated foals has not been determined, we elected to use an equivalent mass (1 g) of GaM. Additionally, treatments were applied weekly to respective plots to simulate ongoing exposure as would occur under field conditions. Groups were compared using linear mixed-effects modeling, and significance was set at a value of *P* < 0.05.

## Supplementary information

Supplementary Materials.

## Data Availability

The datasets generated during and/or analysed during the current study are available from the corresponding author on request.
