# Supplementary material for: A Common Practice of Widespread Antimicrobial Use in Horse Production Promotes Multi-Drug Resistance
Source: Sci Rep. 2020 Jan 22;10:911. doi: 10.1038/s41598-020-57479-9 (PMC6976650; doi:10.1038/s41598-020-57479-9)
Supplement: Supplementary file 1 — Supplementary Materials. [file 41598_2020_57479_MOESM1_ESM.pdf]

Title: A Common Practice of Widespread Antimicrobial Use in Horse Production Promotes Multi- Drug Resistance

Authors: Sonsiray Alvarez-Narvaez, Londa Berghaus, Ellen Ruth Morris, Jennifer Willingham-Lane, Nathan Slovis, Steeve Giguère, Noah Cohen

Supplementary materials legend

S1. A Number of ASVs use in the analysis per treatment group and time-point. **B** Box plots showing the alpha diversity indices for the observed amplicon sequence variants (ASVs) pre- and post- treatment for the two different treatment groups gallium maltolate (GaM) and macrolide and rifampin (MaR) and the untreated control group (Con). Asterisk denotes significance ($p < 0.05$). Box centerlines, edges, whiskers, and points signify the median, interquartile range (IQR), 1.5× IQR, and >1.5× IQR, respectively.

S2. Table contains the top 20 taxa used to perform the GIT microbiome composition analysis.

S3. Box plots show **A** total RPKM per sample, **B** total number of genes per sample. The alpha diversity indices for the observed amplicon sequence variants (ASVs) pre- and post- treatment for the 2 treatment groups gallium maltolate (GaM) and macrolide and rifampin (MaR) and the untreated control group (Con). Asterisk denotes significance ($p < 0.05$). Box centerlines, edges, whiskers, and points signify the median, interquartile range (IQR), 1.5× IQR, and >1.5× IQR, respectively.

S4. Antimicrobial resistance gene abundance (RPKM counts) and diversity (gene counts) differed significantly among groups ($p<0.005$) and time-points for aminoglycosides, bacitracin, glycopeptides, macrolides, phenicols and tetracyclines. Data were analyzed using linear mixed-effects models with fixed effects of treatment, time, and their interactions, and foal nested within farm modeled as a random effect. Post hoc comparisons among groups were made using the method of Sidak.[85] Letters indicate significance within treatment (pre- vs. post- treatment).($p<0.05$).

S5. Antibiotic resistance mechanisms differed significantly among groups ($p<0.005$) and timepoints for aminoglycosides, bacitracin, glycopeptides, macrolides, phenicols and tetracyclines. Data were analyzed using linear mixed-effects models. Letters indicate significance within treatment (pre- vs. post- treatment), numbers between treatments (macrolides and rifampin [MaR] vs. gallium maltolate [GaM] vs. Control [Con]). ($p<0.05$).

S6. Set of scripts used in this study

S7. MaR treatment increased the proportion of fecal macrolides-resistant and rifampin-resistant *Enterococcus spp.* and the number of foals carrying them. Bar chart indicates the percentage of resistant (black) and susceptible (grey) *Enterococcus spp.* to **A** macrolides, **B** rifampin, and the number of foals that carry **C** macrolide-resistant and **D** rifampin-resistant *Enterococcus spp.* Letters indicate significance within treatment (pre- vs. post-treatment), numbers between treatments (macrolides and rifampin [MaR] vs. gallium maltolate [GaM] vs. Control [Con]). ($p<0.05$).

S8. Macrolide and tetracycline class-specific antimicrobial genes differed significantly among treatment groups of foals, consistent with our phenotypic susceptibility testing results. Antimicrobialresistance genes identified in *Enterococcus* spp. RPKM (**A**) and number (**B**) by treatment group (healthy untreated (Control), subclinical pneumonia treated with gallium maltolate (GaM), or subclinical pneumonia treated with macrolide plus rifampin (MaR)). Values with different superscripted letters within resistance to a given class of antimicrobial (*i.e.*, macrolides or tetracycline) differ significantly.

Fig. S1

A

	Pre	Post
**Control**	549,574	529,091
**GaM**	529,355	542,436
**MaR**	532,287	512,614

B

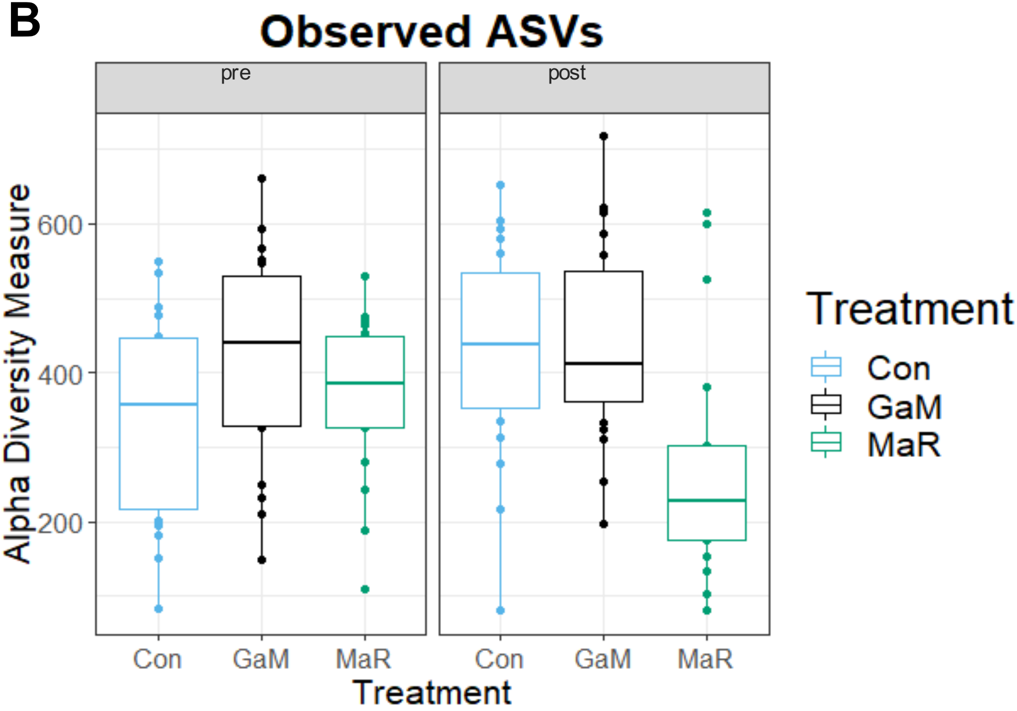

Kingdom	Phylum	Class	Order	Family	Genus
Bacteria	Proteobacteria	Gammaproteobacteria	Enterobacteriales	Enterobacteriaceae	Escherichia/Shigella
Bacteria	Fusobacteria	Fusobacteriia	Fusobacteriales	Fusobacteriaceae	Fusobacterium
Bacteria	Firmicutes	Clostridia	Clostridiales	Ruminococcaceae	Ruminococcaceae_UCG-005
Bacteria	Bacteroidetes	Bacteroidia	Bacteroidales	Prevotellaceae	Alloprevotella
Bacteria	Bacteroidetes	Bacteroidia	Bacteroidales	Bacteroidaceae	Bacteroides
Bacteria	Verrucomicrobia	Verrucomicrobiae	Verrucomicrobiales	Verrucomicrobiaceae	Akkermansia
Bacteria	Verrucomicrobia	WCHB1-41	NA	NA	NA
Bacteria	Bacteroidetes	Bacteroidia	Bacteroidales	Prevotellaceae	Prevotella_6
Bacteria	Fusobacteria	Fusobacteriia	Fusobacteriales	Fusobacteriaceae	Fusobacterium
Bacteria	Bacteroidetes	Bacteroidia	Bacteroidales	Bacteroidaceae	Bacteroides
Bacteria	Bacteroidetes	Bacteroidia	Bacteroidales	Bacteroidaceae	Bacteroides
Bacteria	Actinobacteria	Actinobacteria	Actinomycetales	Actinomycetaceae	Arcanobacterium
Archaea	Euryarchaeota	Methanomicrobia	Methanomicrobiales	Methanocorpusculaceae	Methanocorpusculum
Bacteria	Firmicutes	Clostridia	Clostridiales	Ruminococcaceae	NA
Bacteria	Bacteroidetes	Bacteroidia	Bacteroidales	Prevotellaceae	Prevotellaceae_UCG-004
Bacteria	Verrucomicrobia	Verrucomicrobiae	Verrucomicrobiales	Verrucomicrobiaceae	Akkermansia
Bacteria	Firmicutes	Clostridia	Clostridiales	Ruminococcaceae	NA
Bacteria	Firmicutes	Clostridia	Clostridiales	Lachnospiraceae	NA
Bacteria	Verrucomicrobia	WCHB1-41	NA	NA	NA
Bacteria	Verrucomicrobia	Verrucomicrobiae	Verrucomicrobiales	Verrucomicrobiaceae	Akkermansia

Fig. S3

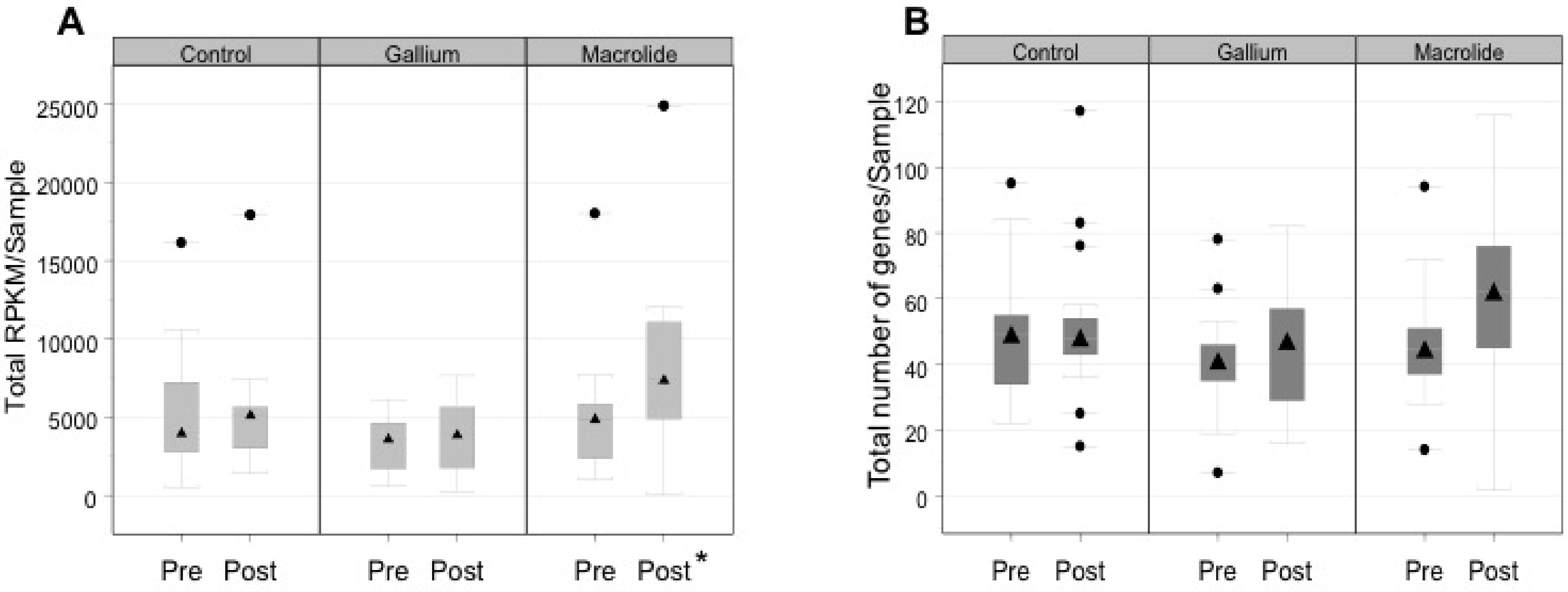
A
Control
Gallium
Macrolide
Total RPKM/Sample
25000
20000
15000
10000
5000
0
Pre
Post
Pre
Post
Pre
Post *
B
Control
Gallium
Macrolide
Total number of genes/Sample
120
100
80
60
40
20
0
Pre
Post
Pre
Post
Pre
Post

S4.

Aminoglycosides: The number of genes and RPKM associated with aminoglycoside resistance increased significantly for the MaR group but not the other groups.

Treatment group	Pre Mean (95% CI)	Post Mean (95% CI)
No. of genes		
Control	1.9 (1.2 to 2.7)[a]	2.0 (1.3 to 2.7)[a]
Gallium	1.1 (0.2 to 2.1)[a]	1.4 (0.1 to 1.9)[a]
Macrolide	1.6 (0.7 to 2.6)[a]	3.0 (2.0 to 4.0)[b]
RPKM		
Control	176.1 (57.4 to 294.7)[a]	161.3 (66.1 to 256.5)[a]
Gallium	63.8 (0.0 to 206.5)[a]	57.1 (0 to 191.7)[a]
Macrolide	151.4 (7.4 to 295.5)[a]	310.0 (172.3 to 447.6)[b]

Bacitracin: There was not a significant increase in the number of genes associated with bacitracin resistance for any of the treatment groups (data not shown). However, the number of RPKM associated with aminoglycoside resistance genes increased significantly for the MaR group but not the other groups.

Treatment group	Pre Mean (95% CI)	Post Mean (95% CI)
RPKM		
Control	346.1 (231.1 to 461.0)[a]	236.6 (106.0 to 367.2)[a]
Gallium	239.2 (76.5 to 401.9)[a]	284.6 (99.9 to 469.3)[a]
Macrolide	312.1 (147.5 to 476.8)[a]	614.9 (426.7 to 803.2)[b]

Glycopeptides: The number of genes and RPKM associated with glycopeptide resistance increased significantly for the MaR group but not the other groups.

Treatment group	Pre Mean (95% CI)	Post Mean (95% CI)
No. of genes		
Control	10.9 (9.0 to 12.9)[a]	10.9 (8.2 to 13.6)[a]
Gallium	8.7 (5.9 to 11.5)[a]	9.4 (5.6 to 13.2)[a]
Macrolide	11.0 (8.2 to 13.8)[a]	15.0 (11.2 to 18.9)[b]
RPKM		
Control	755.4 (507.9 to 1,002.8)[a]	654.6 (319.7 to 989.4)[a]
Gallium	522.1 (175.9 to 868.4)[a]	568.8 (95.2 to 1,042.3)[a]
Macrolide	822.7 (471.7 to 1,173.7)[a]	1,509.9 (1,029.4 to 1,990.5)[b]

S4.

Macrolides: The number of genes and RPKM associated with macrolide resistance increased significantly for the MaR group but not the other groups. RPKM data had to be transformed to meet distributional assumptions of the model (*viz.*, log10 + 1, because of 0 values).

Treatment group	Pre Mean (95% CI)	Post Mean (95% CI)
No. of genes		
Control	5.6 (4.3 to 6.9)[a]	5.1 (3.3 to 6.9)[a]
Gallium	4.3 (2.5 to 6.1)[a]	4.6 (2.1 to 7.2)[a]
Macrolide	4.5 (2.7 to 6.3)[a]	7.3 (4.7 to 9.8)[b]
RPKM		
Control	1.352 (0.889 to 1.815)[a]	1.437 (0.911 to 1.964)[a]
Gallium	0.915 (0.260 to 1.571)[a]	0.973 (0.228 to 1.717)[a]
Macrolide	0.981 (0.318 to 1.644)[a]	1.907 (1.148 to 2.665)[b]

Phenicol: The number of genes and RPKM associated with phenicol resistance increased significantly for the MaR group but not the other groups. RPKM data had to be transformed to meet distributional assumptions of the model (*viz.*, log10 + 1, because of 0 values).

Treatment group	Pre Mean (95% CI)	Post Mean (95% CI)
No. of genes		
Control	0.2 (0 to 0.5)[a]	0.3 (0.0 to 0.6)[a]
Gallium	0.1 (0 to 0.5)[a]	0.2 (0 to 0.7)[a]
Macrolide	0.4 (0.0 to 0.7)[a]	1.0 (0.5 to 1.5)[b]
RPKM		
Control	0.212 (-0.124 to 0.546)[a]	0.283 (-0.114 to 0.679)[a]
Gallium	0.136 (-0.272 to 0.545)[a]	0.261 (-0.300 to 0.822)[a]
Macrolide	0.414 (0.000 to 0.829)[a]	1.209 (0.639 to 1.778)[b]

Tetracyclines: The number of genes and RPKM associated with tetracycline resistance increased significantly for the MaR group but not the other groups.

Treatment group	Pre Mean (95% CI)	Post Mean (95% CI)
No. of genes		
Control	3.0 (2.1 to 3.9)[a]	3.2 (2.1 to 4.2)[a]
Gallium	2.4 (1.2 to 3.5)[a]	2.5 (1.0 to 3.9)[a]
Macrolide	2.6 (1.4 to 3.8)[a]	4.5 (3.0 to 6.0)[b]
RPKM		
Control	239.3(114.3 to 364.3)[a]	259.4 (110.5 to 408.3)[a]
Gallium	172.3 (16.6 to 328.0)a	188.7 (0 to 399.3)[a]
Macrolide	262.0 (104.1 to 419.0)[a]	516.3 (302.5 to 730.2)[b]

S5.

Aminoglycosides: The RPKM data were log10-transformed to meet distributional assumptions of models. The number of genes and RPKM for aminoglycoside O-phospho-transferases and O-nucleotidyl-transferases increased significantly for the MaR group but not the other groups. Other tested mechanisms such as aminoglycoside inactivating proteins, efflux pumps, N-nucleotidyl-transferases, or efflux regulators did not differ significantly among groups (data not shown).

Gene Category	Pre-treatment	Post-treatment
No. of O-phosphotransferases		
Control	0.3 (0.0 to 0.5)[a,b]	0.1 (-0.2 to 0.4)[a]
GaM	0.1 (-0.2 to 0.4)[a]	0.0 (-0.4 to 0.4)[a]
MaR	0.1 (-0.2 to 0.4)[a]	**0.4 (0.1 to 0.8)[b]**
RPMK of O-phospho-transferases		
Control	0.26 (0.01 to 0.51)[a]	0.14 (-0.17 to 0.45)[a]
GaM	0.08 (-0.27 to 0.43)[a]	0.00 (-0.44 to 0.44)[a]
MaR	0.05 (-0.31 to 0.40)[a]	**0.52 (0.08 to 0.97)[b]**
No. of O-nucleotidyl-transferases		
Control	0.1 (-0.1 to 0.3)[a]	0.1 (-0.1 to 0.3)[a]
GaM	0.1 (-0.1 to 0.4)[a]	0.1 (-0.2 to 0.4)[a]
MaR	0.1 (-0.2 to 0.3)[a]	**0.6 (0.3 to 0.9)[b]**
RPMK of O-nucleotidyl-transferases		
Control	0.13 (-0.10 to 0.36)[a]	0.18 (-0.11 to 0.46)[a]
GaM	0.06 (-0.26 to 0.38)[a]	0.05 (-0.36 to 0.46)[a]
MaR	0.08 (-0.25 to 0.40)[a]	**1.02 (0.61 to 1.43)[b]**

S5.

Bacitracin: The number of genes and RPKM for ABC transporters were significantly increased after treatment the MaR group but not after other treatments. Neither RPKM nor gene number increased significantly for undecaprenyl pyrophosphate phosphatase (data not shown).

Gene Category	Pre-treatment	Post-treatment
No. of ABC transporters		
Control	0.8 (0.6 to 1.0)[a,1]	0.6 (0.3 to 0.8)[a,1]
GaM	0.8 (0.3 to 1.4)[a,1]	0.6 (0.3 to 0.9)[a,1]
MaR	0.7 (0.4 to 1.0)[a,1]	**0.9 (0.5 to 1.2)[a,2]**
RPKM of ABC transporters		
Control	250.49 (145.08 to 355.89)[a]	185.80 (63.40 to 308.20)[a]
GaM	184.89 (35.96 to 333.83)[a]	208.31 (35.20 to 381.41)[a]
MaR	232.49 (81.72 to 383.26)[a]	**529.46 (353.13 to 705.78)[b]**

Glycopeptides: The number of genes and RPKMs for VanR genes increased significantly after MaR treatment but not after other treatments. RPKMs (but not gene number) increased significantly after MaR treatment for VanP and VanA.

Gene Category	Pre-treatment	Post-treatment
No. of VanP		
Control	2.5 (1.8 to 3.2)[a]	2.8 (1.8 to 3.8)[a]
GaM	1.6 (0.6 to 2.6)[a]	2.2 (0.7 to 3.6)[a]
MaR	2.4 (1.3 to 3.4)[a]	3.6 (2.1 to 5.1)[a]
RPMK of VanP		
Control	99.78 (56.45 to 143.11)[a]	102.85 (41.57 to 164.13)[a]
GaM	50.92 (-10.30 to 112.15)[a]	77.37 (-9.29 to 164.03)[a]
MaR	93.59 (31.52 to 155.66)[a]	**186.38 (98.52 to 274.24)[b]**
No. of VanR		
Control	8.4 (7.0 to 9.8)[a]	8.0 (6.1 to 10.0)[a]
GaM	6.7 (4.8 to 8.7)[a]	7.2 (4.4 to 9.9)[a]
MaR	8.3 (6.3 to 10.3)[a]	**11.1 (8.2 to 13.9)[b]**
RPMK of VanR		
Control	650.25 (441.83 to 858.67)[a]	603.87 (310.24 to 897.50)[a]
GaM	466.97 (172.48 to 761.46)[a]	434.78 (19.5 to 850.03)[a]
MaR	731.04 (432.49 to 1,029.59)[a]	**1,305.10 (884.07 to 1,726.17)[b]**
No. of VanA		
Control	0.1 (-0.2 to 0.3)[a]	0.1 (-0.4 to 0.5)[a]
GaM	0.3 (-0.1 to 0.7)[a]	0.1 (-0.5 to 0.6)[a]
MaR	0.0 (-0.4 to 0.4)[a]	0.4 (-0.2 to 1.0)[a]
RPMK of VanA		
Control	1.0 (-4.8 to 6.7)[a]	0.7 (-7.4 to 8.8)[a]
GaM	0.6 (-7.6 to 8.7)[a]	0.1 (-11.3 to 11.6)[a]
MaR	0.0 (-7.2 to 7.2)[a]	**19.1 (7.5 to 30.8)[b]**

S5.

Macrolides: When appropriate, data were log10-transformed to meet distributional assumptions of models. Post hoc pair-wise comparisons of groups within time and time within group were made using the method of Sidak. Because most foals had values of 0 for macrolide inactivator genes or RPKM, these genes were analyzed using Fisher's exact test for the binary outcome of whether the inactivator gene was present or absent (irrespective of value of gene number or RPKM). The number of genes and RPKMs for macrolide inactivators increased significantly after MaR treatment but not after other treatments. RPKMs matching with efflux pumps and methyltransferase also increased significantly after MaR treatment but not after other treatments.

**Gene Category**	**Pre-treatment**	**Post-treatment**
No. of Efflux		
Control	1.1(0.6 to 1.5)[a]	1.2 (0.6 to 1.8)[a]
GaM	0.8 (0.3 to 1.4)[a]	1.0 (0.1 to 1.9)[a]
MaR	0.8 (0.2 to 1.5)[a]	1.6 (0.7 to 2.5)[s]
RPKM Efflux		
Control	18.90 (6.80 to 52.54)[a,1]	14.35 (4.14 to 49.74)[a,1]
GaM	7.62 (1.79 to 31.31)[a,1]	8.89 (1.53 to 51.58)[a,1]
MaR	8.07 (1.87 to 34.91)[a,1]	**41.57 (7.06 to 244.92)[b,1]**
No. of Methyltransferases		
Control	0.5 (0.0 to 1.1)[a]	0.6 (0.0 to 1.2)[a]
GaM	0.3 (-0.5 to 1.0)[a]	0.2 (-0.7 to 1.0)[a]
MaR	0.3 (-0.4 to 1.1)[a]	1.0 (0.1 to 1.9)[a]
RPMK of Methyltransferases		
Control	3.15 (1.35 to 7.34)[a]	2.58 (1.06 to 6.31)[a]
GaM	1.67 (0.50 to 5.51)[a]	1.41 (0.40 to 5.00)[a]
MaR	1.95 (0.58 to 6.55)[a]	**7.11 (1.98 to 25.45)[b]**
No. of Inactivators		
Control	1/19 (5%)	1/19 (5%)
GaM	0/19 (0%)	0/19 (0%)
MaR	0/18 (0%)	**5/19 (26%)**
	P = 1.0000; Fisher's exact test	**P = 0.0388; Fisher's exact test**
RPMK of Inactivators		
	Present Before	Present After
Control	1/19 (5%)	0/19 (0%)
GaM	0/19 (0%)	0/19 (0%)
MaR	0/19 (0%)	**5/19 (26%)**
	P = 1.0000; Fisher's exact test	**P = 0.0028; Fisher's exact test**

S5.

Phenicol: The number of genes and RPKM for acetyltransferases were significantly increased after MaR treatment but not after other treatments. Neither RPKM nor gene number increased significantly for the RNA methyltransferases (data not shown).

Gene Category		Pre-treatment	Post-treatment
No. of acetyltransferases			
	Control	0.1 (-0.1 to 0.3)[a]	0.1 (-0.2 to 0.4)[a]
	GaM	0.1 (-0.4 to 0.4)[a]	0.1 (-0.3 to 0.5)[a]
	MaR	0.2 (-0.1 to 0.6)[a]	**0.8 (0.4 to 1.3)[b]**
RPKM of acetyltransferases			
	Control	1.49 (0.74 to 3.02)[a]	1.97 (0.77 to 5.04)[a]
	GaM	1.25 (0.46 to 3.39)[a]	1.37 (0.36 to 5.16)[a]
	MaR	1.87 (0.68 to 5.13)[a]	**10.28 (2.67 to 39.66)[b]**

Tetracyclines: The number of genes and RPKM for tetracycline riboproteinases were significantly increased after MaR treatment but not after other treatments. Tetracycline inactivating genes or efflux pumps did differed significantly among groups (data not shown).

Gene Category		Pre-treatment	Post-treatment
No. of ribonucleoproteases			
	Control	1.9 (1.4 to 2.4)[a,b]	1.9 (1.3 to 2.5)[a]
	GaM	1.3 (0.6 to 2.1)[a]	1.5 (0.7 to 2.4)[a]
	MaR	1.5 (0.7 to 2.2)[a]	**2.7 (1.8 to 3.6)[b]**
RPMK of ribonucleoproteases			
	Control	110.81 (69.94 to 151.67)[a]	108.52 (55.34 to 161.70)[a]
	GaM	65.32 (7.58 to 123.06)[a]	73.42 (-1.80 to 148.63)[a]
	MaR	75.40 (16.88 to 133.92)[a]	**215.74 (139.32 to 292.15)[b]**

S6. Set of scripts used in this study

```
## Microbiome demultiplex coding##
#!/usr/bin/env perl
#
# Written by Kranti Konganti.
# Copyright 2018. Kranti Konganti
#
# Licensed under: https://creativecommons.org/licenses/by-nc-sa/4.0/
#
# Please cite / acknowledge the use of this script as follows:
#
# We used custom perl script to demultiplex samples without quality filtering.
# This script checks for the presence of barcode sequences from the metadata
# index file supplied and demultiplexes the reads into F and R respectively.

use strict;
use warnings;
use Bio::SeqIO;
use Bio::Seq::Quality;

if ($#ARGV < 3) {
  die "\nUsage: $0 Read_1_FASTQ Read_2_FASTQ Barcode_FASTQ Indices_TXT_FILE\n\n";
}

my $platform = "sanger";

my $read_1_fastq_obj = Bio::SeqIO->new(-file => $ARGV[0],
                                       -format => 'fastq',
                                       -variant => $platform);

my $read_2_fastq_obj = Bio::SeqIO->new(-file => $ARGV[1],
                                       -format => 'fastq',
                                       -variant => $platform);

my $bc_fastq_obj = Bio::SeqIO->new(-file => $ARGV[2],
                                                       -format => 'fastq',
                                                       -variant => $platform);

open(I, "<$ARGV[3]") || die "\nCannot open Indices Text File with Index in first column and Sample Name in second column: $!\n\n";


print "\nReading $ARGV[0] ...\n";
my %index_reads1;
while (my $seq_in = $read_1_fastq_obj->next_seq) {
  my $seq_id = $seq_in->id;
  my $seq_desc = $seq_in->desc;
  my $seq_qual = $seq_in->qual;
  my $seq = $seq_in->seq;

  $index_reads1{$seq_id} = "$seq|@$seq_qual|$seq_desc";
}
$read_1_fastq_obj->close;

print "\nReading $ARGV[1] ...\n";

my %index_reads2;
while (my $seq_in = $read_2_fastq_obj->next_seq) {
  my $seq_id = $seq_in->id;
```

```
    my $seq_desc = $seq_in->desc;
    my $seq_qual = $seq_in->qual;
    my $seq = $seq_in->seq;

    $index_reads2{$seq_id} = "$seq|@$seq_qual|$seq_desc";
}
$read_2_fastq_obj->close;

print "\nReading $ARGV[2] ...\n";

my %barcodes;
while (my $seq_in = $bc_fastq_obj->next_seq) {
    my $seq_id = $seq_in->id;
    my $seq_desc = $seq_in->desc;
    my $seq_qual = $seq_in->qual;
    my $seq = $seq_in->seq;

    push @{$barcodes{$seq}}, $seq_id;
}
$bc_fastq_obj->close;


print "\nReading $ARGV[3] ...\n";

while (my $line = <I>) {
    next if ($line =~ m/^#/);
    chomp $line;
    my ($barcode, $sample) = split/\t/, $line;

    my $new_f_name = $sample . '_' . $barcode . '_F.fq';
    my $new_r_name = $sample . '_' . $barcode . '_R.fq';

    my $new_f = Bio::SeqIO->new(-file => ">$new_f_name",
                                                    -format => 'fastq');

    my $new_r = Bio::SeqIO->new(-file => ">$new_r_name",
                                                    -format => 'fastq');

    #my @ids = @{$barcodes{$barcode}};

    foreach my $id (@{$barcodes{$barcode}}) {
            if (exists $index_reads1{$id} && $index_reads2{$id}) {
                my ($read_1_seq, $read_1_qual, $read_1_desc) = split/\|/, $index_reads1{$id};
                my ($read_2_seq, $read_2_qual, $read_2_desc) = split/\|/, $index_reads2{$id};

                my $read1_obj = get_bioseq_qual_obj($id, $read_1_desc, $read_1_qual, $read_1_seq);
                my $read2_obj = get_bioseq_qual_obj($id, $read_2_desc, $read_2_qual, $read_2_seq);

                $new_f->write_seq($read1_obj);
                $new_r->write_seq($read2_obj);
            }
    }

    $new_f->close;
    $new_r->close;
}

close I;
```

```
# Subroutine to construct Bio::Seq::Quality object
sub get_bioseq_qual_obj {
    my $id = shift;
    my $desc = shift;
    my $qual = shift;
    my $seq = shift;

    my $fq_seq = Bio::Seq::Quality->new(-id => $id,
                                        -desc => $desc,
                                        -qual => $qual,
                                        -seq => $seq
                );
    return $fq_seq;
}
```

##Microbiome analysis Rscripts##

```
#Used the 2 websites below to help with and guide data analsysis.
##https://www.bioconductor.org/help/course-materials/2017/BioC2017/Day1/Workshops/Microbiome/MicrobiomeWorkflowII.html#different_ordination_projections
##https://benjjneb.github.io/dada2/tutorial.html
library(dada2); packageVersion("dada2") #1.8.0; R verison - 3.5.1
path <- "C:/Users/ellen/Desktop/AllSeqs_20Aug18"
#path <- "C:/Users/ellen/Desktop/STAMPS_2018/FoalMicrobiota_StampsRunThrough/AllSeqs_20Aug18"
list.files(path)

# Sort ensures forward/reverse reads are in same order
fnFs <- sort(list.files(path, pattern=".R1.fq"))
fnRs <- sort(list.files(path, pattern=".R2.fq"))
# Extract sample names, assuming filenames have format: SAMPLENAME_XXX.fastq
sample.names <- sapply(strsplit(fnFs, ".R1.fq"), `[`, 1)
View(sample.names)
# Specify the full path to the fnFs and fnRs
fnFs <- file.path(path, fnFs)
fnRs <- file.path(path, fnRs)

plotQualityProfile(fnFs[1])
plotQualityProfile(fnRs[1])
## Changes made to plotQualityProfile command based on the fastq files actually containing the quality reads -- Thanks to Kranti's perl script :) 20Aug18

filt_path <- file.path(path, "filtered") # Place filtered files in filtered/ subdirectory
filtFs <- file.path(filt_path, paste0(sample.names, ".F.filt.fastq.gz"))
filtRs <- file.path(filt_path, paste0(sample.names, ".R.filt.fastq.gz"))

out <- filterAndTrim(fnFs, filtFs, fnRs, filtRs, truncLen = c(240,180), maxN = 0, maxEE = 2, compress = TRUE, matchIDs = TRUE, verbose = TRUE)
head(out)

derepFs <- derepFastq(filtFs, verbose=TRUE)
derepRs <- derepFastq(filtRs, verbose=TRUE)
# Name the derep-class objects by the sample names
names(derepFs) <- sample.names
names(derepRs) <- sample.names

errF <- learnErrors(derepFs, multithread = T)
errR <- learnErrors(derepRs, multithread = T)

plotErrors(errF, nominalQ=TRUE)
```

```
plotErrors(errR, nominalQ=TRUE)
#Parameter learning is computationally intensive, so by default the learnErrors function uses only a subset of the data (the first 1M reads). If the plotted error model does not look like a good fit, try increasing the nreads parameter to see if the fit improves

dadaFs <- dada(derepFs, err=errF, multithread=TRUE)
dadaRs <- dada(derepRs, err=errR, multithread=TRUE)

dadaFs[[1]]
dadaRs[[1]]

mergers <- mergePairs(dadaFs, derepFs, dadaRs, derepRs, verbose=TRUE)
# Inspect the merger data.frame from the first sample
head(mergers[[1]])

seqtab <- makeSequenceTable(mergers)
# dim(seqtab)
table(nchar(getSequences(seqtab))) # checking average read length

#removal of chimeric sequences
seqtab.nochim <- removeBimeraDenovo(seqtab, method = "consensus", multithread=T, verbose=T)
dim(seqtab.nochim)

sum(seqtab.nochim)/sum(seqtab)

#tracking the number of reads that made it through the pipeline
getN <- function(x) sum(getUniques(x))
track <- cbind(out, sapply(dadaFs, getN), sapply(mergers, getN), rowSums(seqtab), rowSums(seqtab.nochim))
colnames(track) <- c("input", "filtered", "denoised", "merged", "tabled", "nonchim")
rownames(track) <- sample.names
head(track)

#assigning taxonomy
taxa <- assignTaxonomy(seqtab.nochim,
"C:/Users/ellen/Desktop/STAMPS_2018/FoalMicrobiota_StampsRunThrough/AllSeqs_20Aug18/silva_nr_v128_train_set.fa.gz",
multithread = T)

taxa.print <- taxa
rownames(taxa.print) <- NULL
head(taxa.print)

#Handoff to phyloseq
library(phyloseq); packageVersion("phyloseq") ##Package version -- 1.24.2
library(ggplot2); packageVersion("ggplot2") ## Package version -- 3.1.0
samples.out <- rownames(seqtab.nochim)

#Creating dataframe regarding dada2 output
##SampleMetadata.txt file includes foalID, treatment group and time
SampleMetadata <- read.table("C:/Users/ellen/Desktop/STAMPS_2018/FoalMicrobiota_StampsRunThrough/SampleMetadata.txt",
header = T, sep "\t")
FoalData <- data.frame(SampleMetadata)
rownames(FoalData) <- samples.out
head(FoalData)

ps_without_tree <- phyloseq(otu_table(seqtab.nochim, taxa_are_rows = F), sample_data(FoalData), tax_table(taxa))
ps_without_tree

######Vizualizing the alpha-diversity############
es1 <- estimate_richness(ps_without_tree, split = T, measures = c("Observed", "Shannon"))
cb_palette <- c("#56B4E9", "#000000","#009E73")
```

```
plot_richness(ps_without_tree, x = "Time", measures = "Observed", color="Time") +
  theme_bw() + facet_grid(. ~ Treatment) + ggtitle("Observed ASVs") +
  theme(plot.title = element_text(hjust = 0.5, size=20, face="bold"), legend.text = element_text(size=16), legend.title =
element_text(size=20), axis.title.x = element_text(size=16), axis.text.x = element_text(size=12), axis.title.y = element_text(size=16),
axis.text.y = element_text(size=12), strip.text.x=element_text(size=16)) +
  geom_boxplot() + scale_color_manual(values = cb_palette, labels = c("Pre", "Post")) +
  scale_x_discrete(labels=c("A" = "Pre", "B" = "Post"))

plot_richness(ps_without_tree, x = "Time", measures = "Shannon", color="Time") +
  theme_bw() + facet_grid(. ~ Treatment) + ggtitle("Shannon Diversity Indices") +
  theme(plot.title = element_text(hjust = 0.5, size=20, face="bold"), legend.text = element_text(size=16), legend.title =
element_text(size=20), axis.title.x = element_text(size=16), axis.text.x = element_text(size=12), axis.title.y = element_text(size=16),
axis.text.y = element_text(size=12), strip.text.x=element_text(size=16)) +
  geom_boxplot() + scale_color_manual(values = cb_palette, labels = c("Pre", "Post")) +
  scale_x_discrete(labels=c("A" = "Pre", "B" = "Post"))

##########Ordination - beta diversity #####################
ord.pcoa.bray <- ordinate(ps_without_tree, method="PCoA", distance = "bray")
evals <- ord.pcoa.bray$values[,1]
plot_scree(ord.pcoa.bray)

plot_pcoabrayord <- plot_ordination(ps_without_tree, ord.pcoa.bray, color = "Treatment", shape = "Time", title = "Bray-Curtis PCoA")
+
  geom_point(size = 4) +
  theme_bw() +
  theme(plot.title = element_text(hjust = 0.5, size = 20, face = "bold")) +
  scale_color_manual(values = cb_palette) +
  scale_shape_discrete(labels = c("Pre", "Post")) +
  coord_fixed(sqrt(evals[2]/evals[1]))
plot_pcoabrayord

##### Phlya heatmaps #####
top10 <- names(sort(taxa_sums(ps_without_tree), decreasing=TRUE))[1:10]
ps.top10 <- transform_sample_counts(ps_without_tree, function(OTU) OTU/sum(OTU)) ## transform ASV counts to relative
abundance
ps.top10 <- prune_taxa(top10, ps.top10)

plot_heatmap(ps.top10, taxa.label = "Phylum", sample.label = "Time", sample.order = "Time", low = "Yellow", high = "Red") +
  facet_grid(. ~ Treatment) +
  theme(axis.text.x = element_text(size=9, angle = 360), axis.title.x =
          element_text(size=16), axis.title.y = element_text(size=16),
        axis.text.y = element_text(size=11), legend.text = element_text(size=11),
        legend.title = element_text(size=16), strip.text.x=element_text(size=18))


##### Top 20 Phyla Stacked Bar Plot #####
top20 <- names(sort(taxa_sums(ps_without_tree), decreasing=TRUE))[1:20]
ps.top20 <- transform_sample_counts(ps_without_tree, function(OTU) OTU/sum(OTU)) ## transform ASV counts to relative
abundance
ps.top20 <- prune_taxa(top20, ps.top20)

plot_bar(ps.top20, x="Time", fill="Phylum") +
  facet_wrap(~Treatment, scales="free_x") +
  theme(axis.text.x = element_text(size=10.5, angle = 360, hjust = 0.5)) +
  scale_x_discrete(labels=c("A" = "Pre", "B" = "Post"))


##### Statistical analysis - alpha diveristy #####
write.table(es1, "C:/Users/ellen/Desktop/AllSeqs_20Aug18/esdata.txt", sep="\t")
```

```
###esdata.txt formated with SampleID, Observed_es values (Observed), Shannon_es values (Shannon), Treatment for foal
(Treatment), and sample collection time point (Time).
##install.packages("nlme")

library(nlme); packageVersion("nlme") ## Package version -- 3.1.137
alpha_data <- read.table(file.choose(), sep = "\t", header = T)
alpha_data$Treatment <- as.factor(alpha_data$Treatment)
Shannon_es <- lme(Shannon ~ Treatment*Time, data = alpha_data, random=~1|ID,na.action=na.exclude)
summary(Shannon_es)

Observed_es <- lme(Observed ~ Treatment*Time, data = alpha_data, random=~1|ID,na.action=na.exclude)
summary(Observed_es)

##### Statistical analysis - beta diveristy #####
library(vegan); packageVersion("vegan") ## Package version -- 2.5-3
library(phyloseq)
pcoa.metadata <- read.table(file.choose(), header=T, sep="\t")
##Same format as the metadata file discussed above -- SampleMetadata.txt file includes foalID, treatment group and time
pcoa.metadata$Time <- factor(pcoa.metadata$Time)

ps.sub.mac <- subset_samples(ps_without_tree, Treatment %in% "Mac")
ps.sub.gam <- subset_samples(ps_without_tree, Treatment %in% "GaM")
ps.sub.con <- subset_samples(ps_without_tree, Treatment %in% "Con")

ps.sub.T1 <- subset_samples(ps_without_tree, Time %in% "A")
ps.sub.T2 <- subset_samples(ps_without_tree, Time %in% "B")

### Bray curtis adnois #########################################
###Macrolide Bray
mac.bray <- phyloseq::distance(ps.sub.mac, method = "bray")
pcoa.m.meta <- pcoa.metadata[ which(pcoa.metadata$Treatment == "M"), ]
adonis(mac.bray ~ Time, data=pcoa.m.meta, by = "terms")

###GaM Bray
gam.bray <- phyloseq::distance(ps.sub.gam, method = "bray")
pcoa.g.meta <- pcoa.metadata[ which(pcoa.metadata$Treatment == "G"), ]
adonis(gam.bray ~ Time, data=pcoa.g.meta, by = "terms")

###Control Bray
con.bray <- phyloseq::distance(ps.sub.con, method = "bray")
pcoa.c.meta <- pcoa.metadata[ which(pcoa.metadata$Treatment == "C"), ]
adonis(con.bray ~ Time, data=pcoa.c.meta)

###Pre-treatment Bray
T1.bray <- phyloseq::distance(ps.sub.T1, method = "bray")
pcoa.t1.meta <- pcoa.metadata[ which(pcoa.metadata$Time == "1"), ]
adonis(T1.bray ~ Treatment, data=pcoa.t1.meta, by = "terms")

###Post-treatment Bray
T2.bray <- phyloseq::distance(ps.sub.T2, method = "bray")
pcoa.t2.meta <- pcoa.metadata[ which(pcoa.metadata$Time == "2"), ]
adonis(T2.bray ~ Treatment, data=pcoa.t2.meta, by = "terms")
```

##Resistome analysis scripts##

```
###TRIMMOMATIC
time java -jar /usr/local/apps/eb/Trimmomatic/0.36-Java-1.8.0_144/trimmomatic-0.36.jar \
PE -threads 4 -phred33 1_S1_L001_R1_001.fastq 1_S1_L001_R2_001.fastq \
1_1_trimmed.fq 1_1_unpaired.fq 1_2_trimmed.fq 1_2_unpaired.fq \
ILLUMINACLIP:/usr/local/apps/eb/Trimmomatic/0.36-Java-1.8.0_144/adapters/TruSeq3-PE.fa:2:30:10 \
LEADING:3 TRAILING:3 SLIDINGWINDOW:4:20 MINLEN:40
```

```
###Read sorting
cat file.fastq | paste - - - - | sort -k1,1 -t " " | tr "\t" "\n" > file_sorted.fastq

###Read pairing
pear -f ./1_cutadapt_R1.fastq -r ./1_cutadapt_R2.fastq -o
/scratch/sa73772/workDir/resistomeanalyzer/Faecal_swabs_raw_data/140128-48430425/paired_reads

###Fastq to fasta conversion
paste - - - - < file.fq | cut -f 1,2 | sed 's/^@/>/' | tr "\t" "\n" > file.fa

###Merging Miseq and Nextseq fasta files
cat S1_Miseq.fa S1_Nextseq.fa > S1.combined.fasta

###Merging CARD and MEGARES .faa databases
cat CARD.faa MEGARES.faa > combined_AMR.faa

###CD-HIT
cd-hit -T 2 -i combined_AMR.faa -o AMR_DB.faa

###Diamond
diamond makedb --in AMR_DB.faa -d nr
diamond blastx -d nr -q s1.combined.fasta -o 1.AMR_matches -f 101

##Resistomeanalyzer##
./resistome \
   -ref_fp AMR_DB.faa \
   -sam_fp  1.AMR_matches.sam \
   -annot_fp annotations.csv \
   -gene_fp gene.tsv \
   -group_fp group.tsv \
   -class_fp class.tsv \
   -mech_fp mech.tsv \
   -t 80
```

Fig. S7

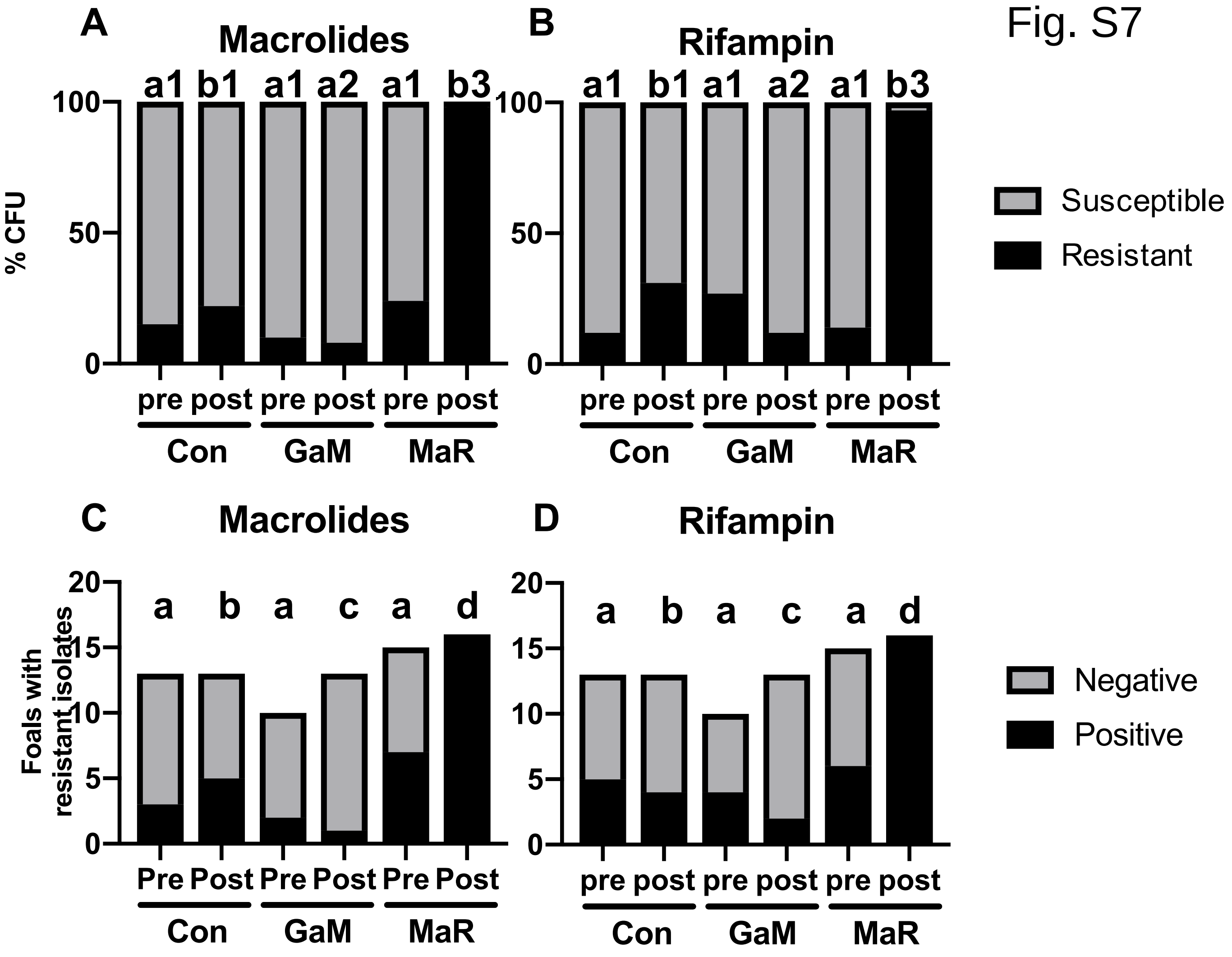
A
Macrolides
a1 b1 a1 a2 a1 b3
100
50
0
% CFU
pre post pre post pre post
Con GaM MaR
B
Rifampin
a1 b1 a1 a2 a1 b3
100
50
0
pre post pre post pre post
Con GaM MaR
Susceptible
Resistant
C
Macrolides
20
15
10
5
0
a b a c a d
Foals with resistant isolates
Pre Post Pre Post Pre Post
Con GaM MaR
D
Rifampin
20
15
10
5
0
a b a c a d
pre post pre post pre post
Con GaM MaR
Negative
Positive

S8

A. RPKM results

**Resistance Gene**	**Time**	**Treatment Groups**		
		**Control(n=19)**	**GaM (n=19)**	**MaR (n=19)**
Macrolide	Pre	8 (22 to 64)[a]	8 (2 to 36)[a]	10 (2 to 42)[a]
	Post	27 (8 to 89)[a]	9 (2 to 50)[a]	**81 (15 to 444)[b]**
Tetracycline	Pre	237 (127 to 347)[a]	170 (13 to 326)[a]	261 (103 to 420)[a]
	Post	257 (111 to 403)[a]	186 (0 to 392)[a]	**514 (304 to 723)[b]**

B. Gene number results

**Resistance Gene**	**Time**	**Treatment Groups**		
		**Control(n=19)**	**GaM (n=19)**	**MaR (n=19)**
Macrolide	Pre	2 (1 to 3)[a]	1(0 to 3)[a]	1 (0 to 3)[a]
	Post	2 (0 to 4)[a]	1 (0 to 3)[a]	3 (1 to 5)[a]
Tetracycline	Pre	3 (2 to 4)[a]	2 (1 to 3)[a]	3 (1 to 4)[a]
	Post	3 (2 to 4)[a]	2 (1 to 4)[a]	**4 (3 to 6)[b]**
